# Artistic Anatomy
*A Manual of Artistic Anatomy, for the use of Sculptors, Painters, and Amateurs. By Robert Knox, M.D., F.R.S.E. London: Renshaw. 1853.


**Published:** 1854-10-01

**Authors:** 


### Art. II.—ARTISTIC ANATOMY.*

About five-and-twenty years ago, Dr. Knox was in the zenith of his fame. He enjoyed, as an anatomist, an European reputation; and was one of the best and most expert Demonstrators attached to any British School of Medicine. His popularity in the north was unbounded. His practical knowledge of the organic structure of the dead body, as it lay stretched on the table before him, while he stood by like a necromancer about to perform some mystical operation; the graceful manner with which he addressed his class; the insinuating tone of voice and smile of fascination (such as artist never yet depicted); the flowery and poetic language with which he described the articulations of the vertebral column, the ligaments of the knee-joint, the anatomy of the axilla; above all the ineffable feeling of scepticism which seemed to thrill through his very scalpel as he unfolded the convolutions of the brain, laid bare its ventricles, and exposed even the commissura mollis to the wondering gaze of the most remote pupil in his gallery; impressed upon the mind the conviction, which has not since been disturbed, that if Kemble or Edmund Kean, whose wonderful

* A Manual of Artistic Anatomy, for the use of Sculptors, Painters, and Amateurs. By Robert Knox, M.D., F.R.S.E. London: Renshaw. 1853.

Shakspearian impersonations we have often watched with thrilling and breathless admiration, were great actors, so also was Knox in his own theatre in Surgeon's-square, the walls of which have resounded with applause as loud, as prolonged, and as enthusiastic as ever shook, or seemed to shake, the Theatre Royal Drury Lane to its foundation, in the most palmy days of dramatic art. Five-and-twenty years have since then passed!

> "Eheu fugaces—Posthume—Posthume
> Labuntur anni."

The University of Jacobus Sextus—ever time-honoured and revered—has, during this *hiatus memorabilis*, lost many of the great lights which then ruled the day; and the school without, but still underneath the shade of the academic walls which Barclay, Gordon, the younger Cullen, Fletcher, Mackintosh, Milligan, adorned with their eminent talent and learning, has undergone we know not how many vicissitudes. There are, however, many bright constellations which still linger in the departing twilight of an age which will hereafter be esteemed memorable; and ever and anon a something crosses our path which brings back to our recollection scenes, incidents, persons, and associations, which abundantly verify the old prediction of Æneas; for they are indeed "pleasant to remember."

Here is, to wit, a small octavo volume on "Artistic Anatomy," by Dr. Knox, from which it would appear that, although his sun may be no longer traversing the ecliptic of the northern hemisphere, it has not yet set, but sheds even now its light upon the difficult and dreary path which the artist—whether sculptor or painter—is doomed to tread before he can reach the temple which he deifies! But we have spoken of Dr. Knox only as an anatomist. He has higher claims. His "Lectures on Comparative Anatomy" were conceived in a truly philosophic spirit, and were listened to with instruction and admiration by pupils who had attended the celebrated courses on the same subjects by Cuvier, Lamarck, and Geoffroy St. Hilaire. His translations, too, of Icedemann, Meckel, and Cloquet's great work on "Descriptive Anatomy"—at a period when these illustrious authorities were little known in this country—conferred upon schools of Medicine, generally, invaluable service. In addition to which his various discoveries in Physiology, which he communicated from time to time to the Royal Society of Edinburgh, and published in desultory scientific journals, have given Dr. Knox a conspicuous and enviable position among the great and original thinkers of his age. There is a class of men so restless, from the prodigality of their powers of invention, that they cannot remain satisfied with the discovery of simple truths. There are, we verily believe, philosophers, who are so superabundantly ingenious, so curiously and inexpressibly desirous of discovering new combinations in the phenomenal world, that they would turn Lord Rosse's telescope itself into a kaleidoscope, rather than survey in a straightforward manner through its gigantic lenses the naked majesty of the heavens, revealing amidst its clustering groups of stars the gaps, so finely described by Humboldt, which appear to open into the illimitable regions of space. It were an easy matter, one might have supposed, for so thorough and so accomplished an anatomist to have shown the relation which exists between the structure of the human body and its representation in the highest works of art, whether in sculpture or painting: but has he accomplished this in the manual before us? Does not Dr. Knox, at every step of his progress, throw in our way some semblance of a paradox—some seemingly incomprehensible proposition which startles us, and challenges contradiction? Before we have even crossed the threshold, in the first paragraph of his Introduction, he tells us that his object "is not merely to teach the artist how to draw or to sculpture the human frame correctly; he has a higher aim." He then continues, and we beg to call attention to *our* italics:—

"The fine arts to which I limit my present view, sculpture and painting, *most unquestionably are not*, as M. Quatremère de Quincy seems to have thought, *merely imitative* arts; such an expression is inapplicable in every sense to the compositions of Michael Angelo, the Cena of Da Vinci; the Cartoons of Raphael, the Apollo and Venus of antiquity, and generally to the inimitable works of the unknown antique sculptors. The Parthenon was *not* the product of an *imitative* mind; no *mechanical-minded Saxon* could have imagined or designed Egyptian Thebes .... *Assured of the soundness of my views, I will yet go further; the rustic scenes of Teniers and Ostade, the landscapes of Cuyp, of Hobbima and Vanderveld, the interiors of Gerard Dow, the compositions of Wouvermans, are no more imitations than the grand conceptions of Raphael, and are as much unlike their modern imitators as Astley's Theatre is to the Coliseum.*"

Here we may well pause, and ask what our learned anatomist really means? The highest conceptions of genius cannot transcend the sphere of our own consciousness. We can disintegrate or combine objects in any way we please; but can never create or imagine an object, the form of which is not already impressed upon the senses. All the heraldic anomalies emblazoned in the Herald's College, our griffons, our sphinxes, our centaurs, our unicorns, our double-headed eagles, what are they more than "combinations of disjointed things"? Proceed higher; what can the finest landscape of Claude, or Salvator Rosa, or the most sublime conception of Poussin, be more than nature herself portrayed under her most lovely, her wildest, or most sublime forms? The "Last Judgment" of Michael Angelo, on the plafond of the Sistine Gallery, when analysed, presents to us clouds, figures, heads, which, in the midst of their majestic array, have their types in nature. Look at the "Last Supper," by Leonardo da Vinci; are not those apostles, sitting round the table of their Blessed Master, in figure, face, and gesture, in every sense human? Are not those hands laid upon the table designed after the very likeness and fashion of the hand as it should be studied by every painter who would faithfully delineate it? Or take the picture of "Our Lady of the Immaculate Conception," by Murillo, the purchase of which from Marshal Soult's collection lately excited such a fervour of contention in Paris. What have we here? A female figure, with "grave, sweet eyes and golden hair," and beautiful features, her hands crossed on her bosom as if in prayer. She is supported on clouds. From her head, as from a sun, radiate streams of light; under her feet are visible the horns of the crescent moon. Beneath the clouds is seen the outline of the globe, on the surface of which a serpent is gliding along. "To those conversant with the mysteries of religious art," says the eloquent artistic critic from whom we have taken this description,* "this picture has a meaning which the uninitiated cannot penetrate. The Virgin is here represented not only as 'Maria purissima sin pecado concebida,' but as the second Eve, whose seed was to bruise the head of the serpent. The painter has endowed her with the attributes of the Woman of the Apocalypse, 'clothed with the sun, having the moon under her feet, and on her head a crown of twelve stars.'" Now, there is not an image in this magnificent composition, with all its veiled, emblematical, and allegorical meaning, that is not borrowed from, and suggested by, imitative art. Still more unfortunate for Dr. Knox's paradox is his reference to the Dutch school of painting: the pictures of Teniers are literally a transcript of living nature; and for the interiors of Dow; a lady once observed to us, in the Louvre, when we were looking at the "Cottage Scene," where the family had assembled at a table, "You might see the threads of the very cloth!" Every artist knows—and indeed Dr. Knox would not have troubled himself to have written this artistic Manual himself if he also did not know—that Art is, and must ever be, strictly imitative, however genius may invest its productions with suggestive representations, and lights and shadows which carry the imagination beyond the dim confines of humanity.

We are next told by Dr. Knox, that "*no mechanical-minded Saxon could have imagined or designed Egyptian Thebes!*" and hereupon he throws at our feet this reproof: "*A taste for the Fine Arts, and of consequence the condition of these arts, is about as low in Britain as it can*

* Edinburgh Review, January, 1853.

*well be.*" Again we pause, and would break a lance with this "learned Theban." What does Dr. Knox mean, not only in this Manual, but in his book on the "*Races of Men*," by disclaiming against what he is pleased to call the "Saxon race"? Who are they? Whence came they? If, as would appear from the context in different portions of his work, Dr. Knox levels these aspersions against the English, as a people, we inform him that his anthropology is at fault! It was very well for Daniel O'Connell and "the Agitators" of Ireland, as they styled themselves, during a period of great political commotion, to declaim against the "Saxon;" but this was adopted as a mere political war-cry, such as conflicting parties always have had recourse to during the excitement of great civil conflicts. The French of yore shouted "*Montjoye;*" the Normans, "*Dieu Aide;*" the Flemish, "*Arras;*" the Augeoines, "*Balie;*" the Bretons, "*Mallon;*" and why should not the Irish in the nineteenth century, in rousing the passions of their Celtic followers, cry out, "the Saxon"? But Dr. Knox is too good an ethnologist to recognise the existence really of any such race, which, according to his own non-transmission theory, must long ago have been as extinct as the Saxon Heptarchy itself! He must know, that the English people are now a-days no more "Saxons" than they are Danes or Normans. But apart from this, if Dr. Knox seriously entertains the opinion concerning Art which he has above expressed, does he not lay himself open to the question whether his information respecting the state of the Fine Arts in this country is sound? Did he never hear of Sir Joshua Reynolds, Morland, Wilkie, Turner, Haydon? If he will walk into Sir John Soane's Museum, in Lincoln's Inn-fields, some fine afternoon, he will there find the pictures of an insignificant personage named William Hogarth, who is supposed ("mechanical-minded Saxon" as he may have been) to have had *some* genius and feeling for his art! If still sceptical, we would invite him to accompany us to Hampton Court, and admire with us (if he have any taste or love for woman) the Beauties of the Court of Charles II. Here, however, as ladies are in the case, we might possibly, albeit the age of chivalry is gone, wax warm, and would in that case challenge him to repair with us to France, not for the purpose of measuring distances and drawing our rapiers, but for that of visiting the charming Palace of Versailles; and there, perambulating the polished floor of its long galleries, we would call upon him to select from the whole range of celebrated portraits, the idolized beauties of France, from the reign of Francis I. to that of Louis XV.; from the host of Vallières, Maintenons, De Pompadours, any pictures equal in artistic excellence to those of Sir Peter Lely and Sir Godfrey Kneller. We could pursue this subject much further if our space permitted; but we cannot dismiss it without reminding Dr. Knox that we have artists now living among us of whose genius any nation might be proud—if genius were limited, which happily it is not, to climes and races. Indeed, we have the triumphant satisfaction of knowing that, so long as David Roberts, Lance,* Stanfield, Danby, Eastlake, Knight, Gordon, Grant, Maclise, Sir Edwin Landseer, Cooper, &c., &c., contribute their annual offerings at the shrine of British Art, our own Royal Academy will every successive year give a flat contradiction to the reckless assertion of Dr. Knox, that the state of the Fine Arts is as low in this country as it can be. Our only astonishment is, that any author attempting to write a Manual for the guidance of the English student in sculpture and painting should so grievously commit himself; but as we proceed, kicking out of our way paradox after paradox which some evil genius must surely have scattered on the path of Dr. Knox, we stumble upon one which our footsteps refuse to pass without some special mark of indignation! What shall we do with it? How handle it? Where throw it? How crush it? We walk round it, like a traveller who meets with some curious monstrosity in the highway, almost afraid to touch it lest it sting him. Surely we were mistaken. We read it again. No! Our vision did *not* deceive us. Here is the very passage:—"Experience," says Dr. Knox—Mark! gentle reader! for we believe fair eyes do sometimes glance over our pages—"Experience had told me *that woman's mind had no real sympathies with the Fine Arts* (!)—*that she does not understand their meaning or their object* (!!) *Nature's landscape itself, whether spread out before her or represented on canvas, she passes heedlessly by* (! ! !) *Her mind is a matter-of-fact mind—delicate, tender, soft; but clear, observing of detail, devoted to the real. To her, next to herself, man is all! Fashion obeys; she commands and creates it—jewels, rich garments, tapestry, gorgeous carpets, display!*" Fie! fie! Dr. Knox. Is it thus you speak of the sex whom you are known so much to idolize, and who, if report speaks truly, you are capable of so greatly fascinating by the sauvity of your manner and the charms of your conversation? We will empanel a jury of ladies to try the question of libel. Were we to do so, we suspect they would pronounce a summary verdict, and that the learned Doctor, before being dismissed from behind the bar, would have to

* In Haydon's "Autobiography," reviewed in a previous number of this Journal, the following entry occurs in his diary: "*I have educated two great artists—Lance and Eastlake.*" Why, we ask, is not this "great" and inimitable artist a Royal Academician, or even an Associate? George Lance is unquestionably unrivalled in this, and we believe in every other country, in his own specialté, and is certainly entitled to have honorary distinction conferred upon him. Apart from his extraordinary artistic talent, he possesses many intellectual, amiable, and estimable moral and social qualities. His admission into the Royal Academy would not only reflect a lustre upon that distinguished body, but give general satisfaction to all admirers of genius and true lovers of British Art.

kneel, and according to an old but not obsolete custom, "sue the mercy of the court."

As we set out with stating, so we repeat, that Dr. Knox is an excellent anatomist, an accomplished physiologist. The anatomical descriptions contained in the work before us are deserving every praise —clear, concise, and graphic. They will be useful to the young artist, who must commence with the study of anatomy, and who should remember that when Michel Angelo wished to begin a statue he made first a paper on the skeleton, afterwards upon another paper the same figure clothed with muscles; in this way he executed the statues of Christ, in the Church of Minerva, at Rome, many of which studies were long preserved. So also Goethe remarked, that "The human form cannot be comprehended merely through seeing its surface; it must be stripped of its muscles, its parts separated, its joints observed, its divisions marked; its action and counter-action, the hidden, the reposing, the foundation of the apparent, searched, if one would really see and imitate what moves as a beautiful inseparable whole in living waves before the eye." Let the young artist treasure up these observations; and whatever taste, or talents, or even genius he may possess, or fancy himself to be endowed with, let him endeavour to acquire a thorough knowledge of the fundamental principles upon which he must work out his conceptions. If the marble from its shapeless block is to be chiselled into a symmetrical, graceful, almost breathing statue, such as might, like the fabled Pygmalion, descend from her enchanted pedestal;—if the blank and naked canvas is to be made to glow with a living picture, the visible impression of all that can be imagined beautiful in earth or sublime in Heaven!—the master-hand that brings into palpable existence such wondrous conceptions must know even mechanically how to deal with the details! There is no Promethean spark lying hidden in the unmodelled clay, waiting to leap into life at the sculptor's touch; there is no magic power, concealed charm-like, in the palette upon which the poor artist must make up and blend his colours, which are of themselves of "the earth earthy;" he must toil, toil, toil. No men ever laboured harder in their vocation than the great artists of antiquity! Talk, indeed, of the mechanical-minded Saxon!—declaim idly and untruly against the present state of British Art,—how can such mistaken views assist the young artist industriously plodding at his easel? It is an old saying, that, "there is no royal road to geometry;" nor can Genius itself, without patient study, master the difficulties which lie at the threshold of every science. Let the student, therefore, persevere. He may rest assured that the deeper the foundations of knowledge, the more secure and perfect will be its superstructure. Let him, therefore, be guided by the practical anatomical details in the Manual before us, which he will find really valuable to him, and not pursue apocryphal opinions which evince more ingenuity than judgment. We have seen, during the last half century, many stars in the ascendant—not a few of which have set; but the brightest, the most refulgent and enduring of any have been those which in the morning of life have risen slowly and steadily.

---

### Art. III.—THE CORRELATION OF PSYCHOLOGY AND PHYSIOLOGY.

